# Predictors and Clinical Outcomes of Level IV Nodal Metastasis in Oral Cavity Squamous Cell Carcinoma: A Retrospective Cohort Study of 578 Node-Positive Patients

**DOI:** 10.7759/cureus.95991

**Published:** 2025-11-03

**Authors:** Sanjay Chandra Das, Harsh Thakran, Sanuja Paul, Prateek Jain, Guru Charan Sahu, Vikram Dilip Kekatpure, Kapila Manikantan, Angotu Jayakrishnasai, Dhiraj Kumar Mahato, Pattatheyil Arun

**Affiliations:** 1 Department of Emergency Medicine, Tata Medical Center, Kolkata, IND; 2 Department of Head and Neck Surgery, Tata Medical Center, Kolkata, IND

**Keywords:** disease free survival (dfs), level iv nodal metastasis, oral cavity squamous cell carcinoma, overall survival (os), skip metastasis

## Abstract

Background

Nodal metastasis in oral cavity squamous cell carcinoma (OCSCC) is associated with poorer prognosis. Limited evidence is available on the predictive factors and prognostic significance of level IV nodal metastasis. This study evaluated patient outcomes by analyzing disease-free survival (DFS) and overall survival (OS) in individuals with level IV nodal involvement.

Methods

The cohort for this retrospective study comprised all patients with node-positive OCSCC who underwent surgery between August 2011 and June 2021. The patients’ demographic and treatment details were abstracted from the electronic medical records. DFS and OS were analyzed using a log-rank test for univariate analysis and the Cox proportional hazards model for multivariate analysis.

Results

Among the cohort of 578 node-positive patients, 27.2% exhibited level IV nodal metastasis. The two-year DFS and OS rates in the level IV nodal metastasis group were 55% and 72.2%, respectively. Patients with level IV involvement had a median age of 53 years (range: 21-85 years), were predominantly male (69.2%), and had a median follow-up of 30.1 months (range: 1-131.9 months). A significantly higher prevalence of level IV nodal metastasis was associated with pathological tumor stage 4b (pT4b) (45.3% vs. 11.7%, p < 0.01), pathological nodal stage 3b (pN3b) (30.6% vs. 29.0%, p = 0.01), larger maximum tumor size (Tmax) (3.36 cm vs. 3.42 cm, p < 0.01), and greater depth of invasion (DoI) (1.44 cm vs. 1.63 cm, p < 0.01).

In univariate analysis, DFS was significantly associated with the primary site, Tmax, DoI, involved margins, lymphovascular invasion (LVI), perineural invasion (PNI), masticator space involvement, extranodal extension (ENE), and level IV nodal metastasis, in addition to pathological tumor stage (pT), pathological nodal status (pN), and overall stage. In multivariate analysis, the primary site, Tmax, involved margins, LVI, and PNI remained independently associated with DFS. For OS, univariate analysis identified the primary site, Tmax, DoI, involved margins, LVI, PNI, bone involvement, and ENE, as well as pT, pN, and overall stage, as significant predictors. Following multivariate adjustment, the primary site, Tmax, involved margins, and PNI emerged as independent predictors of OS.

Conclusion

The two-year DFS and OS rates for 578 patients with node-positive OCSCC were 55% and 72.2%, respectively. Among these patients, 157 (27.2%) exhibited level IV nodal metastasis, with skip metastasis to level IV observed in only 3.2% of cases. Patients with pT4b disease, pN3b disease, larger Tmax, and greater DoI were significantly more likely to have level IV metastasis. In univariate analysis, level IV metastasis was significantly associated with worse DFS. Additionally, primary tongue tumors, larger tumor size, involved surgical margins, and the presence of LVI or PNI were predictive of poorer DFS. For OS, significant predictors included primary tongue origin, larger tumor size, positive margins, and PNI.

## Introduction

Oral cavity cancers are among the most common cancers in India [1]. Chewing betel quid, smoking, and drinking alcohol are the most common and established risk factors [2]. Surgical resection followed by adjuvant radiation or chemoradiation, when indicated, forms the mainstay of treatment [3-5]. Although the treatment of oral cavity squamous cell carcinoma (OCSCC) has improved significantly over time, the outcomes after treatment completion remain poor due to recurrence.

OCSCC has a high risk of lymph node metastases, which also significantly impacts the survival outcomes. Multiple factors, including location, size, thickness, depth of invasion (DoI), and tumor grade, have been reported to contribute to nodal metastasis in OCSCC [6-8]. Level I-III nodes are usually considered the first nodal stations for metastasis; however, skip metastases to level IV have been reported [9]. Minimal literature is available regarding the factors predicting and prognostic implications of level IV nodal metastasis. The aim of this study was twofold: (1) to identify predictors for level IV nodal metastases in OCSCC and (2) to assess the impact of level IV metastasis on overall survival (OS) and disease-free survival (DFS) to offer insightful information on the prognosis and treatment in this cohort of patients, with the goal of enhancing patient care and treatment approaches for these patients.

This article was presented as a poster at the “Future Physicians for Change” Conference, Washington, DC, on 31st May 2024.

## Materials and methods

The cohort for this retrospective study comprised all patients with node-positive OCSCC who underwent surgery between August 2011 and June 2021 at a tertiary care cancer center in Eastern India. Patients with node-negative disease, those who had received prior treatment for head and neck cancer, and those for whom accurate follow-up data could not be retrieved were excluded from the study.

The data for our analysis were sourced from the electronic medical records (EMRs). Patients received adjuvant therapy based on their adverse features, which included radiotherapy with or without chemotherapy. Age, gender, and primary site were abstracted from the EMR. The size of the tumor, grade, margin status, bone invasion, masticator space involvement (MSI), pathological tumor stage (pT), pathological nodal status (pN), lymphovascular invasion (LVI), perineural invasion (PNI), DoI, extranodal extension (ENE), and levels of nodal metastasis were taken directly from postoperative histopathology reports. All patients were discussed in the tumor board, which included head and neck surgeons, radiation oncologists, medical oncologists, and histopathologists. Adjuvant treatment decisions were taken based on the patient’s age, general condition, tumor stage, and other risk factors. Patients receiving adjuvant irradiation received 60 Gy to the tumor bed and involved nodal stations, whereas 54 Gy was delivered to the uninvolved nodal levels, in conventional doses of 2 Gy day⁻¹. Patients receiving adjuvant chemoradiation received weekly cisplatin 40 mg/m^2^ in addition to the irradiation.

The surgery date was used as a reference date for calculating survival data. DFS was defined as the days between the date of surgery and the date of disease recurrence or death, whichever event occurred first. Every attempt was made to confirm the recurrences pathologically. DFS for patients without documentation of disease recurrence or death was censored at the last day of follow-up. OS was defined as the days between the date of primary surgery for OCSCC and the date of death due to any cause. OS for patients without documentation of death was censored at the last date the patient was known to have been alive.

Data analysis was performed using Stata version 14.2 (Stata Corp., College Station, TX, USA). Comparisons of categorical variables between Ipsilateral Level IV-positive and Ipsilateral Level IV-negative groups were performed using the chi-square test when all expected cell counts were ≥5 and Fisher’s exact test when any expected count was <5. Comparisons of continuous variables between groups were performed using Wilcoxon rank sum tests. Kaplan-Meier curves were used to estimate DFS and OS. Comparisons of DFS and OS between groups were performed using log-rank tests. Multivariate Cox proportional hazards models were used to assess the prognostic effects of demographic and tumor characteristics and adjuvant treatment on DFS and OS. Variables with univariate p-values <0.05 were candidates for inclusion in the multivariate models. The Cox proportional hazards model was used for multivariate analysis. The level of statistical significance for all tests was p <0.05, derived from two-tailed tests. Since this is a retrospective study of EMRs that did not involve patients directly, a consent waiver was obtained from the institutional review board vide letter no. EC/WV/TMC/32/24.

## Results

Our study demonstrates a significant association between advanced T and N stages and disease recurrence. Among the 24 patients with pT4b disease, 66.7% (n = 16) experienced recurrence, including nine cases of distant metastasis, four local recurrences, and three nodal recurrences. Similarly, among 75 patients with N3b disease, 52% (n = 39) had recurrence, comprising 21 distant metastases, 11 local recurrences, and seven nodal recurrences. Overall, there were 245 recurrence events and 155 deaths during the study period. The cohort of 578 node-positive patients had a median age of 53 years (range: 21-85 years) and a median follow-up duration of 30.1 months (range: 1-131.9 months). The group included 400 men (69.2%) and 178 women (30.7%). The most common primary tumor site was the tongue (n = 294, 50.86%), followed by the buccal mucosa (n = 284, 49.13%). A total of 157 patients (27.2%) exhibited level IV nodal metastasis, of whom five (3.2%) had no involvement of levels I-III. Demographic and histopathological characteristics are summarized in Table 1.

**Table 1:** Demographic and histopathological factors of patients with OCSCC Data are presented as n (%) of total cohort, N = 578. Statistical comparisons were performed using chi-square (†) or Fisher’s exact (‡) test, as appropriate. Statistical significance was defined as p <0.05. ^†^Chi-square – Gender, primary site, margin status, LVI, PNI, bone involvement, ENE, adjuvant treatment. ^‡^Fisher’s exact – Differentiation, pT stage, pN stage (due to small cell counts <5). IL4: ipsilateral level IV lymph node; LVI: lymphovascular invasion; PNI: perineural invasion; ENE: extranodal extension; pT: pathological tumor size; pN: pathological nodal status; adjuvant RT: adjuvant radiotherapy; adjuvant CTRT: adjuvant chemoradiotherapy; pN3b: pathological nodal stage 3b.

Variable	Category	IL4 Positive, n (%)	IL4 Negative, n (%)	Not Dissected, n (%)	p-Value
Gender^†^	Male	112 (19.4%)	268 (46.4%)	20 (3.5%)	0.459
	Female	45 (7.8%)	127 (22.0%)	6 (1.0%)	
Primary site^†^	Tongue	82 (14.2%)	204 (35.3%)	8 (1.4%)	0.112
	Buccal mucosa	75 (13.0%)	191 (33.0%)	18 (3.1%)	
Differentiation^‡^	Well	3 (0.5%)	8 (1.4%)	1 (0.2%)	0.052 (Fisher)
	Moderate	105 (18.2%)	224 (38.8%)	20 (3.5%)	
	Poor	49 (8.5%)	163 (28.2%)	5 (0.9%)	
Margin status^†^	Free (>5 mm)	89 (15.4%)	290 (50.2%)	16 (2.8%)	0.002
	Close (1-4.9 mm)	57 (9.9%)	86 (14.9%)	10 (1.7%)	
	Involved (<1 mm)	11 (1.9%)	19 (3.3%)	0 (0.0%)	
LVI^†^	Present	108 (18.7%)	251 (43.4%)	14 (2.4%)	0.258
	Absent	49 (8.5%)	144 (24.9%)	12 (2.1%)	
PNI^†^	Present	95 (16.4%)	247 (42.7%)	11 (1.9%)	0.121
	Absent	62 (10.7%)	148 (25.6%)	15 (2.6%)	
Bone involvement^†^	Present	41 (7.1%)	89 (15.4%)	6 (1.0%)	0.661
	Absent	116 (20.1%)	306 (52.9%)	20 (3.5%)	
ENE^†^	Present	81 (14.0%)	192 (33.2%)	5 (0.9%)	0.008
	Absent	75 (13.0%)	204 (35.3%)	21 (3.6%)	
pT stage^‡^	pT1	4 (0.7%)	26 (4.5%)	4 (0.7%)	0.001 (Fisher)
	pT2	39 (6.7%)	96 (16.6%)	13 (2.2%)	
	pT3	39 (6.7%)	117 (20.2%)	4 (0.7%)	
	pT4a	51 (8.8%)	127 (22.0%)	5 (0.9%)	
	pT4b	24 (4.2%)	29 (5.0%)	0 (0.0%)	
pN stage^‡^	pN1	52 (9.0%)	114 (19.7%)	13 (2.2%)	0.018 (Fisher)
	pN2a	6 (1.0%)	34 (5.9%)	0 (0.0%)	
	pN2b	22 (3.8%)	71 (12.3%)	7 (1.2%)	
	pN2c	1 (0.2%)	12 (2.1%)	0 (0.0%)	
	pN3a	1 (0.2%)	0 (0.0%)	0 (0.0%)	
	pN3b	75 (13.0%)	164 (28.4%)	6 (1.0%)	
Adjuvant treatment^†^	Follow-up	14 (2.4%)	19 (3.3%)	1 (0.2%)	0.372
	Adjuvant RT	73 (12.6%)	202 (34.9%)	10 (1.7%)	
	Adjuvant CTRT	70 (12.1%)	174 (30.1%)	15 (2.6%)	

**Figure 1:**
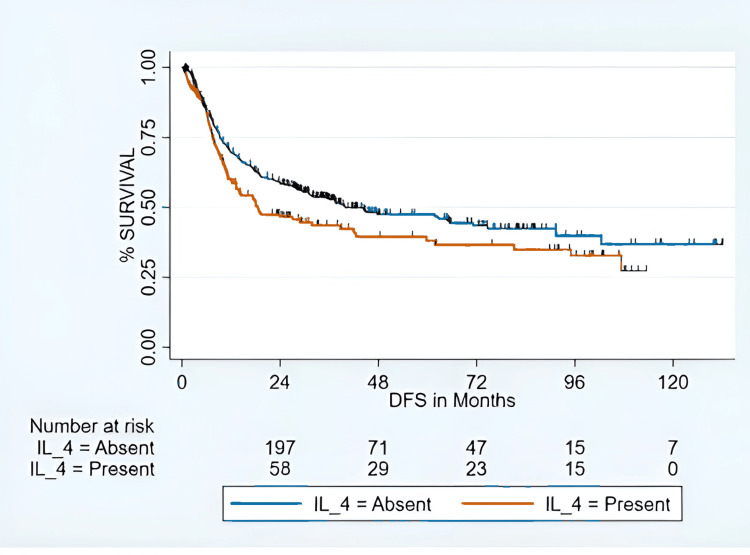
DFS for level IV nodal metastasis Kaplan-Meier curves showing DFS for patients with and without ipsilateral level IV nodal metastasis (IL-4). Group comparisons were performed using log-rank test. Numbers at risk at each time point are displayed below the curves. Statistical significance is defined as p <0.05. DFS: disease-free survival; IL-4: ipsilateral level IV lymph node.

**Figure 2:**
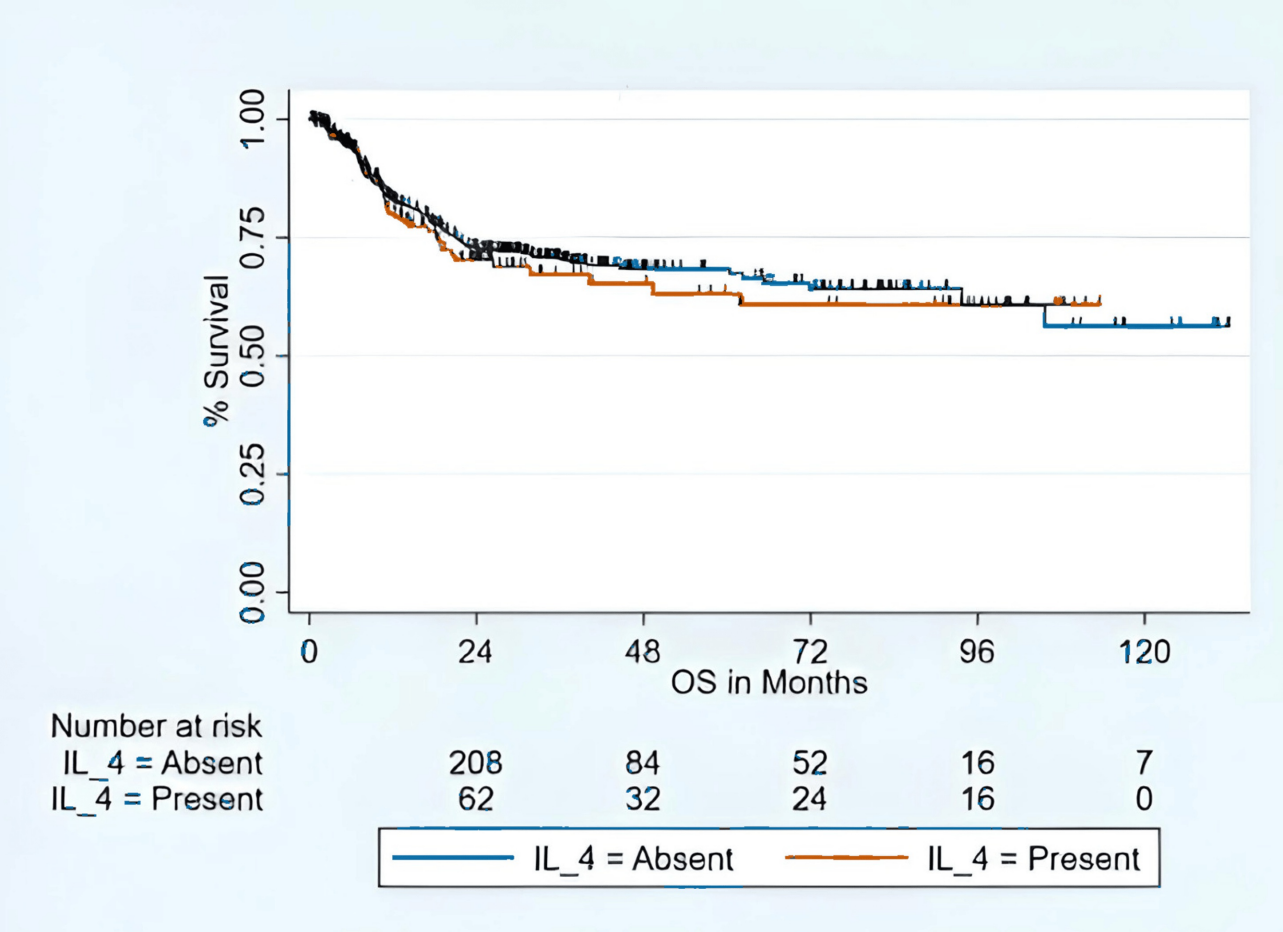
OS for level IV nodal metastasis Kaplan-Meier curves showing OS for patients with and without ipsilateral level IV nodal metastasis (IL-4). Group comparisons were performed using log-rank test. Numbers at risk at each time point are displayed below the curves. Statistical significance is defined as p <0.05. OS: overall survival; IL-4: ipsilateral level IV lymph node.

Level IV metastasis refers to the spread of tumor cells to the lower-jugular group of cervical lymph nodes located in the lower part of the neck along the internal jugular vein. According to the American Joint Committee on Cancer (AJCC) 8th edition TNM staging system, most patients presented with advanced-stage disease, with two (0.3%) in stage I, one (0.2%) in stage II, 132 (22.8%) in stage III, and 443 (76.6%) in stage IV. The incidence of level IV metastasis increased with advancing tumor and nodal stage and with adverse histopathologic features. Level IV metastasis was observed in 11.8% of pT1 tumors and 45.3% of pT4b tumors (p < 0.01). Similarly, the frequency of level IV involvement was 29.0% in pN1 and 30.6% in pathological nodal stage 3b (pN3b) disease (p = 0.01). ENE occurred in 29.1% of cases with level IV metastasis compared with 25.3% without (p < 0.01). Tumors with close (36.8%) or involved (36.7%) surgical margins showed a higher incidence of level IV metastasis than those with free margins (22.5%) (p < 0.01). The 24-month DFS rate for the cohort was 55% (Figure 1), and the 24-month OS rate was 72.2% (Figure 2).

Additionally, among patients with level IV nodal metastasis, 70 (12.11%) underwent adjuvant chemoradiotherapy, 73 (12.6%) received radiotherapy, and 12 (2.07%) were managed with follow-up alone. From the entire cohort of 578 patients, MSI was observed in 52 patients, and bone invasion in 136 patients. Patients with pT4b disease (45.3% vs. 11.7%, p < 0.01), N3b disease (30.6% vs. 29%, p = 0.01), higher T sizes (3.36 vs. 3.42 cm, p = 0.02), and a greater DoI (1.44 vs. 1.63 cm, p < 0.01) showed predilection for nodal IV metastasis.

In univariate analysis, the primary site (hazard ratio (HR) 0.62, 95% CI (0.49-0.78), p < 0.01), maximum T size (Tmax; HR 1.23, CI (1.14-1.39), p < 0.01), DoI (HR 1.33, CI (1.19-1.49), p < 0.01), involved margins (HR 3.31, CI (2.16-5.08), p < 0.01), LVI (HR 1.56, CI (1.21-2.03), p < 0.01), PNI (HR 1.95, CI (1.51-2.53), p < 0.01), MSI (HR 1.97, CI (1.39-2.79), p < 0.01), ENE (HR 1.78, CI (1.41-2.25), p < 0.01), and level IV nodal metastasis (HR 1.31, CI (1.02-1.70), p = 0.04) affected DFS apart from the pT, pN, and overall stage. In multivariate analysis, the primary site (HR 0.60, CI (0.45-0.80), p < 0.01), Tmax (HR 1.17, CI (1.04-1.33), p = 0.01), involved margins (HR 2.16, CI (1.36-3.44), p < 0.01), LVI (HR 1.38, CI (1.04-1.84), p = 0.03), and PNI (HR 1.58, CI (1.18-2.10), p < 0.01) significantly predicted DFS (Table 2).

**Table 2:** Factors predicting DFS Data are presented as HR with 95% CI. Univariate and multivariate analyses were performed using Cox proportional hazards regression. Corresponding z-statistics are reported for each variable. Variables with p <0.05 in univariate analysis were entered into the multivariate model. The study cohort included 578 patients, with 285 (49.3%) DFS events. Statistical significance was set at p <0.05. DFS: disease-free survival; HR: hazard ratio; CI: confidence interval; Tmax: maximum tumor size; DoI: depth of invasion; LVI: lymphovascular invasion; PNI: perineural invasion; MSI: masticator space involvement; ENE: extranodal extension; pT: pathological tumor size; pN: pathological nodal status; z-value: Wald z-statistics.

Serial No.	Factor	Univariate HR (95% CI)	z-Value	p-Value	Multivariate HR (95% CI)	z-Value	p-Value
1	Primary site (buccal mucosa)	0.62 (0.49-0.78)	-4.02	<0.001	0.60 (0.45-0.80)	-3.53	<0.001
2	Tmax	1.23 (1.14-1.33)	5.29	<0.001	1.17 (1.04-1.33)	2.62	0.009
3	DoI	1.33 (1.19-1.49)	5.08	<0.001	-	-	-
4	Involved margins	3.31 (2.16-5.08)	6.07	<0.001	2.16 (1.36-3.44)	3.31	<0.001
5	LVI	1.95 (1.15-3.53)	2.54	0.011	1.38 (1.04-1.84)	2.21	0.028
6	PNI	1.95 (1.15-3.53)	2.16	0.031	1.58 (1.18-2.10)	3.06	0.002
7	MSI	1.97 (1.39-2.79)	3.66	<0.001	-	-	-
8	ENE	1.78 (1.41-2.25)	4.99	<0.001	-	-	-
9	Level IV nodal metastasis	1.31 (1.02-1.70)	2.05	0.041	-	-	-
10	pT2	1.66 (0.79-3.50)	1.27	0.201	-	-	-
11	pT3	3.11 (1.56-6.41)	3.25	0.001	-	-	-
12	pT4a	3.22 (1.56-6.63)	3.44	<0.001	-	-	-
13	pT4b	5.03 (2.33-10.83)	4.95	<0.001	-	-	-
14	pN2a	1.36 (0.83-2.22)	1.18	0.240	-	-	-
15	pN2b	1.39 (0.95-2.02)	1.73	0.084	-	-	-
16	pN2c	0.93 (0.37-2.30)	-0.08	0.940	-	-	-
17	pN3a	3.78 (1.52-27.30)	2.40	0.016	-	-	-
18	pN3b	2.23 (1.67-2.97)	2.99	0.003	-	-	-
19	Overall stage	1.89 (1.39-2.58)	3.98	<0.001	-	-	-

Univariate analysis for factors predicting OS, primary site (HR 0.60, CI (0.44-0.84), p < 0.01), Tmax (HR 1.35, CI (1.22-1.49), p < 0.01), DoI (HR 1.42, CI (1.23-1.64), p < 0.01), involved margins (HR 4.42, CI (2.62-7.44), p < 0.01), LVI (HR 1.85, CI (1.28-2.68), p < 0.01), PNI (HR 2.67, CI (1.82-3.90), p < 0.01), bone involvement (HR 1.62, CI (1.15-2.29), p < 0.01), and ENE (HR 2.22, CI (1.60-3.07), p < 0.01) showed statistical significance, apart from the pT, pN, and overall stage. In multivariate analysis, the primary site (HR 0.61, CI (0.41-0.90), p = 0.02), Tmax (HR 1.28, CI (1.06-1.47), p < 0.01), involved margins (HR 2.60, CI (1.48-4.58), p < 0.01), and PNI (HR 1.93, CI (1.27-2.91), p < 0.01) significantly predicted OS (Table 3). The primary site significantly influenced survival outcomes. Patients with buccal mucosa primaries had better DFS and OS compared to those with tongue primaries.

**Table 3:** Factors predicting OS Data are presented as HR with 95% CI. Univariate and multivariate analyses were performed using Cox proportional hazards regression, with z-statistics reported. Variables significant at p <0.05 in univariate analysis were entered into the multivariate model. N = 578 patients, with 153 (26.4%) OS events. Statistical significance was set at p <0.05. OS: overall survival; Tmax: maximum tumor size; DoI: depth of invasion; LVI: lymphovascular invasion; PNI: perineural invasion; ENE: extranodal extension; pT: pathological tumor size; pN: pathological nodal status; z-value: Wald z-statistics.

Serial No.	Factor	Univariate HR (95% CI)	z-Value	p-Value	Multivariate HR (95% CI)	z-Value	p-Value
1	Primary site (buccal mucosa)	0.60 (0.44-0.84)	-3.06	0.002	0.61 (0.41-0.90)	-2.36	0.018
2	Tmax	1.35 (1.22-1.49)	5.85	<0.001	1.28 (1.06-1.47)	2.70	0.007
3	DoI	1.42 (1.23-1.64)	4.72	<0.001	-	-	-
4	Involved margins	4.42 (2.62-7.44)	4.58	<0.001	2.60 (1.48-4.58)	3.26	0.001
5	LVI	1.85 (1.28-2.68)	3.34	0.001	-	-	-
6	PNI	2.67 (1.82-3.90)	4.72	<0.001	1.93 (1.27-2.91)	3.12	0.002
7	Bone involvement	1.62 (1.15-2.29)	2.77	0.006	-	-	-
8	ENE	2.22 (1.60-3.07)	4.89	<0.001	-	-	-
9	pT2	5.28 (0.71-39.22)	1.59	0.110	-	-	-
10	pT3	14.66 (2.02-106)	3.04	0.002	-	-	-
11	pT4a	16.45 (2.27-118.74)	3.09	0.002	-	-	-
12	pT4b	17.40 (2.30-131.36)	3.03	0.002	-	-	-
13	pN2a	2.14 (1.08-4.25)	2.19	0.029	-	-	-
14	pN2b	2.11 (1.22-3.64)	2.70	0.007	-	-	-
15	pN2c	2.02 (0.70-5.81)	1.03	0.302	-	-	-
16	pN3a	1.12 (0.59-2.02)	0.37	0.712	-	-	-
17	pN3b	3.49 (2.24-5.43)	5.29	<0.001	-	-	-
18	Overall stage	3.14 (1.89-5.20)	4.25	<0.001	-	-	-

Of the total cohort of 578 patients with node-positive OCSCC, 157 patients were IL-4-positive. Of these IL-4-positive patients, 45.9% (n = 72) had recurrence of their disease, with 35 developing distant metastasis, 27 local recurrences, and 10 nodal failures.

## Discussion

​​​​​Historically, OCSCC has been treated with surgery alone. The current standard of care comprises surgical resection with neck dissection followed by adjuvant radiation or chemoradiation [10]. However, data are lacking regarding the predictors and outcomes of level IV node-positive OCSCC within the Indian population. The purpose of this study was thus to estimate the predictors and impact of level IV metastasis in OCSCC at our institution in the period August 2011 to June 2021.

Male patients were most affected in our study, comprising 69.2% (400 cases) of the study population. Other studies from our region and other parts of the Indian subcontinent corroborated this trend of increased prevalence of OCSCC in male patients [11]. Although most of the patients in the Western world with cancer of the oral cavity are observed at an older age [12], the average age of the patients in our study was 53, which exceeds the age of 43.3 quoted by Patel et al. [13]. OCSCC contains several malignancies, and lymph node involvement differs depending on the primary site.

Research has shown that oral tongue squamous cell carcinoma represents the most frequent primary location for OCSCC, which is consistent with our findings, as the tongue accounted for a slightly higher proportion of cases compared to the buccal mucosa (50.9% vs. 49.1% overall, and 52.2% vs. 47.8% among patients with level IV nodal metastasis) [14]. In Altuwaijri et al.’s systematic review, lower-level dissection was suggested for carcinoma of the tongue, perhaps due to the tongue containing an extensive lymphatic network [14]. Patients with buccal mucosa primaries had a better DFS and OS in univariate and multivariate analyses.

Most studies suggest that primary neck dissections should be confined to the upper levels only, due to the low incidence of metastasis in the lower levels (level IV and beyond) and the challenges, along with potential complications, associated with including those levels. Due to the proximity of the thoracic duct, especially on the left, dissection of lower cervical levels, particularly level IV, increases the risk of chyle leak [15]. Diaphragmatic paralysis could result from damage to the nearby phrenic nerve [16].

With a cumulative five-year survival rate of around 50%, which decreases to 30% in advanced stages, these cancers contribute significantly to morbidity and mortality [10]. The DFS of 55% and OS of 72.2% at two years in node-positive OCSCC patients compared favorably with previously published outcomes. Based on surveillance, epidemiology, and end results (SEER) data, Marchiano et al. reported a five-year DFS of only 30.6% for level IV metastases compared to 42% at levels I-III [17], while Hasegawa et al. described a three-year OS of 27.3% (synchronous) and 57.1% (metachronous) in patients with level IV disease [18].

Skip metastasis refers to the condition in which OCSCC bypasses levels I, II, and III and moves directly to level IV. In the systematic review by Altuwaijri et al., the incidence of skip metastasis to level IV nodal metastasis was low, reaching 8.5% [14]. Our study aligns with these findings, showing that skip metastasis to level IV occurs in only 3.2% of OCSCC cases.

Among IL4-positive patients, recurrence rates were notably high across several high-risk pathological subgroups. Our study shows a significant impact of higher T and N stages on recurrence. Our data, demonstrating frequent recurrence (66.7% in pT4b; 52% in N3b) in IL4-positive OCSCC patients, are consistent with wider evidence that nodal metastasis outside level III significantly deteriorates prognoses. A SEER study (n = 8,281) found five-year disease-specific survival of only 30.6% when level IV nodes were involved, significantly lower than the 42% for levels I-III (p < 0.0001) [17]. Likewise, a Japanese cohort (n = 291) discovered that three‑year OS fell to 27% with level IV/V metastasis at the initial presentation [18]. These results confirm our observed unfavorable outcomes in high-risk IL4-positive patients and emphasize the need for total neck dissection to level IV.

A recent 2025 Nature study of patients with pT4a gingivobuccal squamous cell carcinoma reported that tumors with a DoI greater than 10 mm had significantly poorer survival, with the median OS reduced by almost half compared with tumors showing superficial invasion. In multivariable analysis, deep DoI, ENE, and positive resection margins independently predicted adverse outcomes. The authors concluded that DoI may have greater prognostic importance than bone or skin invasion in advanced oral cavity carcinoma [19]. These findings parallel our results, in which increased DoI, ENE, and close or involved margins were significantly associated with level IV nodal metastasis, suggesting that the same pathological factors that worsen survival also contribute to more extensive lower-neck spread in OCSCC.

Challenges and limitations

The limitations of this study include its retrospective design and relatively small sample size. The other limitation of the study is that we only included patients from a single clinical site in our sample. Although this helps maintain consistency in diagnostic standards and procedures, the results may be inapplicable to patients from diverse geographical regions, healthcare environments, or demographic backgrounds. More prospective trials and randomized controlled trials (RCTs) must be conducted across different locations and diverse patient groups to improve the generalizability of the findings.

## Conclusions

In this study of 578 patients with node-positive oral squamous cell carcinoma, the two-year DFS and OS rates were 55% and 72.2%, respectively. Level IV nodal metastasis occurred in 157 patients (27.2%), while true skip metastasis to this level was uncommon, seen in only 3.2%. The presence of level IV disease was closely linked to advanced T and N stages, larger tumor size, and greater DoI, and it was associated with poorer DFS. Additionally, primary tongue tumors, involved or close surgical margins, and the presence of LVI or PNI were also significant predictors of reduced DFS and OS. Although infrequent, level IV metastasis reflects a more aggressive tumor biology. Early recognition of these high-risk features may support more tailored adjuvant therapy and closer postoperative surveillance, ultimately improving long-term outcomes for patients with node-positive oral cavity cancers.
